# Supplementary material for: Comparative genomics analysis of the MYB gene family in barley: preliminary insights into evolution and biological function in Blue Qingke
Source: PeerJ. 2024 Dec 2;12:e18443. doi: 10.7717/peerj.18443 (PMC11619697; doi:10.7717/peerj.18443)
Supplement: Supplemental Information 1 [file peerj-12-18443-s001.docx]

>HvMYB53

ATGGGGAGGATGAGGAAGGAAGGAGTGAAGAGAGGGGCATGGACTAGCAAGGAGGACGAAACCTTGGCTTCCTACGTCAAGGCGCATGGCGAAGGCAGATGGAACGAAGTCCCCCTAAGAGCTGGTCTTCGGCGGTGCGGCAAGAGCTGTCGGCTGCGTTGGCTGAACTACCTTCGGCCTAACGTCAAACGGGGAAATATATCCAACGATGAGGAGGAGATCATCGTCAGGCTCCACGCCCTCCTTGGCAACAGGTGGTCCATCATCGCTGGCAGGTTGCCTGGTCGAACAGATAACGAAATCAAGAACTACTGGAACAGCACCCTTAGCCGGAAGGTGCTTCCCGCACCACATTCCGCCACGAGGATGGTAGCCACGCCCGACACCTCCGCCGGCTCTGGATGTTACACAGAGACGTCGGCAGCGCTGTGTAACTGTGGACCTCGTACAGAGGCGTCGGCAGCGCTGCCGGCCGCGCTGTGGGCGCCAAAGCCTGTGAGGTACACGGGGCTCCCCTTCTTTGGCCGGGATAAGTCACTGCGGTCGCCTGTTGCGGAGACGCGAACCGTGGCCAACGGGGATAACTGCAGCCGCAGCAGCTCCGTAACATCGGAGTTCCCGGCTATACCGCCCTCGTTAGACGGTGGCGACTGGATGGACGAAGTGAGAGCCTTGGAATCGTTTCTCGAATCCGACGAAGACTGGGTAAACTCTGTGGACATGCCGCTATAA

>HvMYC

ATGGCGCTATCAGCTCCTCCCAGTCAGGAACAGCCGTCGGGGAAGAAATTCGGCTATCATCTCGCTGCTGCTGTGAGGAGCATCAACTGGACTTATGGTATATTTTGGTCTATTTCCGCCAGCCCGCGCCCAGGCCACTCCTCAGTTCTGACGTGGAAGGATGGGTTCTACAACGGCGAGATAAAGACAAGAAAGATTACCGGCTCGACCACTCCGGAGTTTACCCCGGACGAGCGCGTCATGCACAGAAGCAAGCAACTGAGGCAGCTCTACGAATCGCTCTTGCCCGGCAACTCCGACCAACGGGCAAGGCGATGCGCCGCATCACTGTCACCGGAGGATCTCGGGGACGGCGAGTGGTATTACACCATAAGCATGACTTACAAGTTCCACCTTAATCAAGGGTTGCCAGGCAAAAGCTTTGCGAGCAATCAATATGTTTGGTTGTGCAACGCTCAGAACGCAAGCACAAGAACTTTTCCGCGCGCACTCTTAGCAAAGACTGCCTCTATTCAGACAATCGTCTGCATCCCCTTCATGGGCGGCGTGCTTGAGCTCGGAACGTTGGACCAGGTTTTGGAGGACTCCAGCATGGTGAAGCGGATCAGCACGTCTTTCTGGGAGCTGCACTTGCCGGCATCCTTGGAGTCGAAGGATCGGAGCTCCAGCACACAAGCAAAAGAAACCAGGGAGGCCACCGACATCATCTTGTTCGAGGACTTCGACCACAGCGACACAGTTGACGGGATGATCTCTGAGCAAAGGGAGGTCCAGTGCCCGTCCAACGTCAATCTCGAACGCCTCACAACGCAGATGGACGAGTTCCACAGCCTTCTTGGGGGACTGGACGTGCACCCTGTCGAAGAAAGATGGATCATTGACGAGCCCTGTGAGTTTATGTCTTCCCCGGAAGTGGCGCCGGCTATGAATATGCCGAGCACCACCGATGTCGTCGTCACTTCAAGTAGGTCCGAAGGCTCTCGTCCATCCTGCTTCACAGCGTGGAAGGGATCATGCGAGTCGAAACGCGTGGCTGGCCAGGTCGTTGGGGAGTCACAGAAGTTGCTGAATAAAGTTGTGACTGGTGGTGCATGGGCGAGCAATTATGGCGGTGGAACCATGGTGAGAGCTCCAGAAAGTAAGAACAAGACCCATGTCATGGCAGAGAGAAGACGCCGGGAGAAGCTCAACGAGATGTTCCTGGTTCTCAAGTCACTAGTCCCTTCCATTCACAAGGTAGACAAAGCATCCATCCTCACAGAAACGATAGGCTATCTCAGAGAACTGAAGCAAAGGGTAGATCAGCCAGAATCTAGCCGGTCACCGTCTGACCCAAAAGAACTCACAGGACGGAGCCGAAGCCATGTCGTCGGCGCTAGGAAGAAGATAGTCTCAGCCGGATCCAAGAGGAAGTCTCCAGGGTTGGAGAGCCCGAGCAATGTCGTGAACGTGACGGTGCTGGACAAGGTGGTGCTGTTGGAGGTAAAGTGCCCGTGGAAGGAGCTGCTGATGACACAAGTATTTGACGCTATCAAGAGCCTCTATCTGGATGTTGTCTCCGTGCACGCATCCACATCGGGTGGCCGTCTTGACCTCAAGATACGGGCTAATCAGCAGCTTGCGGCTGGTGCTGCTATCGTGGCACCTGGGGTAATCACTGAAGCGCTTCAGAGAGCTCTATAG
